# Risk factors for PDA ligation and neurodevelopmental outcomes at corrected 18–24 months in very low birth weight infants

**DOI:** 10.1186/s12887-024-04860-6

**Published:** 2024-05-31

**Authors:** Sol Kim, Sae Yun Kim, Ju-Ae Shin, Young-Ah Youn

**Affiliations:** Department of Pediatrics, Seoul St. Mary’s Hospital, College of Medicine, The Catholic University of Korea, 222 Banpo-daero, Seocho-gu, Seoul, 06591 Republic of Korea

**Keywords:** Patent ductus arteriosus, Surgical ligation, Very low birth weight, Neurodevelopmental impairment

## Abstract

**Background:**

Patent ductus arteriosus (PDA) is commonly encountered morbidity which often occurs as up to 60% of extremely preterm infants born at < 29 weeks gestational age (GA).

**Purpose:**

This study aims to assess the clinical risk factors associated with PDA ligation among very low birth weight infants (VLBWI) and evaluate the neurodevelopmental outcomes of those who underwent PDA ligation.

**Methods:**

A total of 540 VLBWI were initially diagnosed with PDA in our 50-bed level IV NICU at Seoul St. Mary’s Hospital, The Catholic University of Korea, between January 2015 and June 2023. Among these 540 VLBWI with PDA, only 221 had consistent hemodynamically significant (hs) PDA which required intervention including fluid restriction, medical treatment, or surgical ligation. In this study, only those 221 VLBWI with hsPDA who underwent neurodevelopmental assessment at corrected 18–24 months of age were included in this study analysis.

**Results:**

Out of 221 VLBWI diagnosed with hemodynamically significant (hs) PDA, 133 (60.2%) underwent PDA ligation, while the remaining 88 (39.8%) had their hs PDAs closed either medically or with fluid restriction. The mean gestational age and birth weight were significantly lower in PDA ligation group compared to no PDA ligation group (27.02 ± 2.17 vs. 27.98 ± 2.36, 907.31 ± 258.36 vs. 1006.07 ± 283.65, *p* = 0.001, 0.008). Resuscitation including intubation at delivery and intraventricular hemorrhage (IVH) (grade ≥ 3) were significantly higher in PDA ligation group. The clinical outcomes in the PDA ligation group revealed significantly worse oucomes compared to the no PDA ligation group. Both resuscitation, including intubation at delivery, and IVH (grade ≥ 3), consistently exhibited an increased risk for PDA ligation in a multivariable logistic regression analysis. Concerning neurodevelopmental outcomes, infants who underwent PDA ligation demonstrated significantly lower cognitive scores. However, only IVH (grade ≥ 3) and PVL were consistently associated with an increased risk of abnormal neurodevelopment at the corrected age of 18–24 months.

**Conclusion:**

In our study, despite the consistent association between cognitive developmental delays in VLBWI who underwent PDA ligation, we observed that sicker and more vulnerable VLBWIs, specifically those experincing severe IVH, consistently exhibited an increased risk for both PDA ligation and abnormal neurodevelopment at the corrected age of 18–24 months.

## Introduction

Patent ductus arteriosus (PDA) is frequently encountered morbidity in neonatal intensive care unit (NICU) which occurs inversely with gestational age. It occurs in up to 40–60% of very low birth weight infants (VLBWI) during the first few weeks of life [1] and more than 60% of extremely preterm infants born at < 29 weeks gestational age (GA). (2–3) Recent controlled observational studies have not found evidence of benefit from PDA ligation and have associated PDA ligation with an increased incidence of bronchopulmonary dysplasia [4–7] ROP, (4–5) and neurodevelopmental impairment (NDI) at 18–24 month follow-up. (4–5) Along with increasing emphasis on more conservative approach to PDA in preterm infants, surgical PDA ligation is reserved for those requiring consistently increase in respiratory support when medical treatments have either failed or were contraindicated [8]. Further, recent data suggest that both medical and surgical treatments for the PDA are associated with poor outcomes. (9–10) As a result, some clinicians may opt to avoid treating PDA because they believe that it can spontaneously close without intervention in the majority of premature infants, even after discharge [11]. The noninferiority of nonintervention was additionally reported to be attributable for closing PDA, especially in infants born at 23 to 26 weeks’ gestation [12]. However, some critically ill infants with an increasing need for ventilatory support or developing symptoms of necrotizing enterocolitis may require closure of their hemodynamically significant (hs) PDA. Amidst the controversies surrounding PDA treatment and the growing trend of avoiding PDA ligation and prophylactic intervention due to concerns regarding lack of benefit or adverse outcomes of surgical management, our aim was to assess the clinical risk factors associated with PDA ligation among VLBWI and evaluate the neurodevelopmental outcomes of those who underwent PDA ligation.

## Materials and methods

### Study design

Out of 603 VLBWI born, a total of 540 were initially diagnosed with PDA in our 50-bed level IV NICU at Seoul St. Mary’s Hospital, The Catholic University of Korea, between January 2015 and June 2023. Among these 540 VLBWI with PDA, only 221 had persistent hs PDA which required intervention, including fluid restriction, medical treatment, or surgical ligation. Consequently, a total of 319 VLBWI were excluded from the analysis: 300 VLBWI had spontaneous closure of their PDAs without intervention ( not hs PDA), 3 died, 6 were diagnosed with congenital anomalies, and 10 were lost to long-term follow-up at corrected 18–24 months of age.

The clinical outcomes relating to hs PDA, hospital morbidities and neurodevelopmental outcomes were evaluated. VLBWI were screened for PDA within the first week of life, and their diagnoses were confirmed with a two-dimensional echocardiogram. If hs PDA was found in the first week of assessment, serial echocardiogram and close monitoring were continued weekly until hs PDA was resolved. The contraindication for ibuprofen treatment was the various clinical contraindications (e.g., bleeding tendency with positive DIC, severe intraventricular hemorrhage (IVH) ≥3, thrombocytopenia with platelet count less than 50 × 10^3^/µL and acute renal failure (ARF), etc.) or because pharmacological interventions failed after 2 or more cycles of medications used. The surgical ligation was proceeded for VLBWI who were assessed to be at least moderate-stage hs PDA (stage C3) based on the criteria developed by McNamara and Sehgal [13]. No infant received prophylactic treatment for PDA in this study period. Throughout the study, oral or intravenous (IV) ibuprofen was prescribed at 10 mg/kg of ibuprofen as an initial dose, followed by two additional doses of 5 mg/kg on consecutive days [14]. 

The VLBWI with complex congenital heart disease or right-to-left or bidirectional PDA shunting were excluded from this study. The exclusion criteria for this study included VLBWI with no hs PDA, major congenital anomalies or in life-threatening conditions.

Surgical ligation of PDA was routinely performed at the patient’s bedside in the NICU, and all ligations were performed by one experienced pediatric thoracic and cardiovascular surgeon using a metal clip through the third or fourth intercostal space after posterolateral thoracotomy. The mean surgical time was an average duration of 27 min.

### Data definitions

VLBWI were defined as all preterm babies who were born at less than 32 weeks of gestation or born weighing less than 1,500 g. Small for gestational age (SGA) was defined by birth weight below the 10th percentile for GA and sex [15]. Sepsis was determined based on positive blood culture results: early onset sepsis was if the positive result was confirmed within 7 days after birth and late onset sepsis was diagnosed after 7 days. Severe brain injury was defined as intraventricular hemorrhage grade 3 or 4 on cranial ultrasonography [16]. Bronchopulmonary dysplasia (BPD) was diagnosed if oxygen use exceeding 0.21% was still needed at a corrected gestational age of 36 weeks. Necrotizing enterocolitis (NEC) was defined as stage II or higher according to Bell’s classification [17]. Severe IVH was defined as IVH ≥ 3 ; active bleeding with enlarged ventricles, and the grade designation was based on Drs. Papile and Levene’s classification criteria [18]. Severe retinopathy of prematurity was defined as neovascular tufts found posterior to the ridge and classified higher than stage 3 according to the International Classification of Diseases for ROP or requiring treatment. (19–20) Full feeding was defined as the attainment of enteral feeding exceeding > 100 ml/kg/day, at which point the peripherally inserted central catheter could be removed.

The requirement for informed consent from the study subjects was waived by the IRB of by the Ethics Committee of Seoul St. Mary’s Hospital due to the retrospective study design. All methods were conducted in accordance with applicable guidelines and regulations of the Ethics Committee of Seoul St. Mary’s Hospital.

### Statistical analysis

Continuous variables are expressed as means ± standard deviation (SD) and categorical variables are expressed as numbers and proportions. Pearson’s χ2 test was used to analyze categorical variables between two groups. Student’s t test was used to compare continuous variables between groups. Statistical analysis was conducted using SPSS software, version 26.0 (IBM, Corp., Armonk., NY). A *P*-value of < 0.05 was considered statistically significance.

## Results

**Fig. 1:**
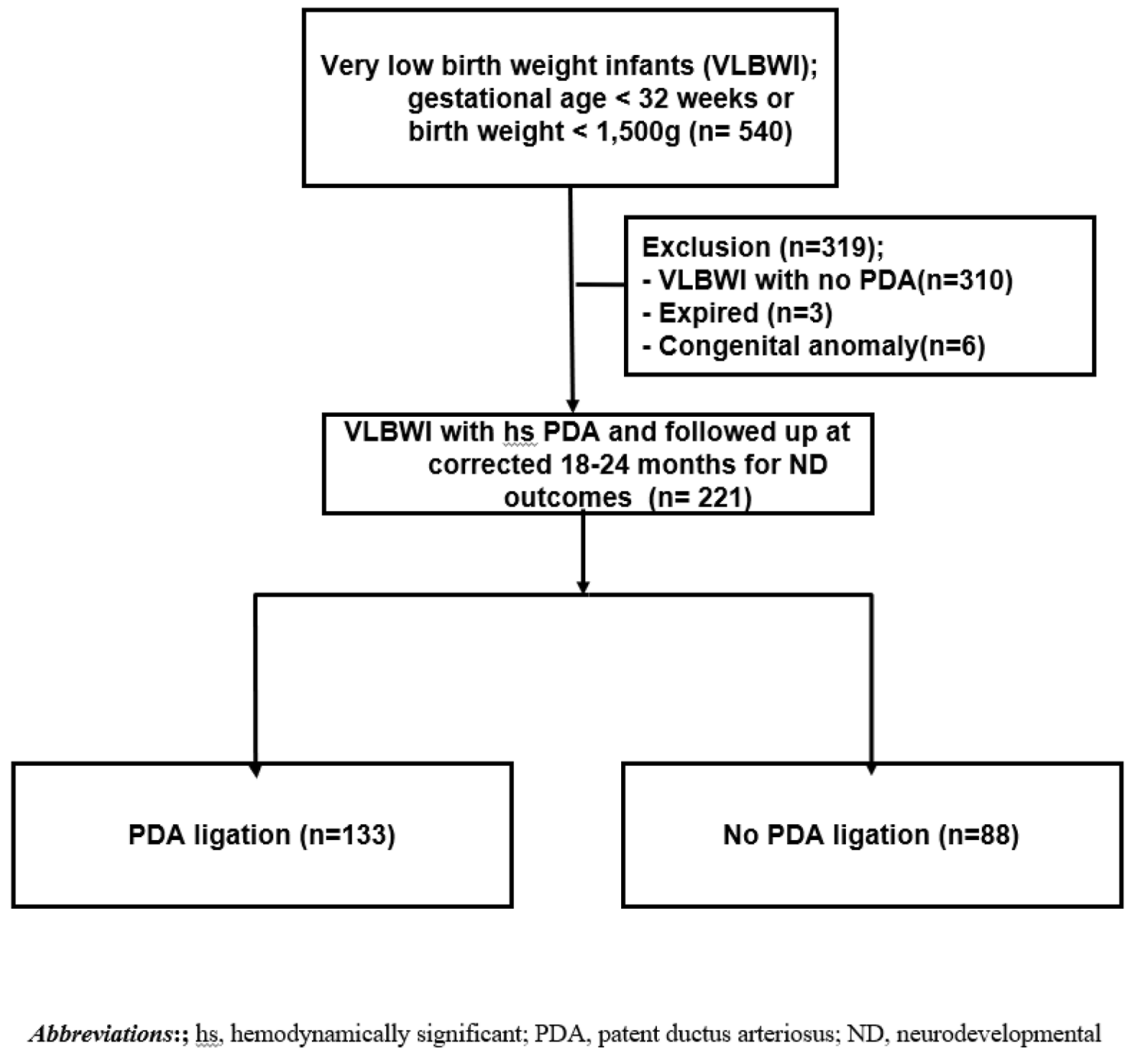
The study flow diagram

In this study, only those VLBWI with hsPDA who underwent neurodevelopmental assessment at corrected 18–24 months of age were included for analysis. Out of 221 VLBWI diagnosed with persistent hs PDA, 133 (60.2%) underwent PDA ligation, while the remaining 88 (39.8%) had their hs PDAs closed either spontaneously or medically. (Fig. 1).

The mean gestational age and birth weight were significantly lower in PDA ligation group compared to no PDA ligation group (27.02 ± 2.17 vs. 27.98 ± 2.36, 907.31 ± 258.36 vs. 1006.07 ± 283.65, *p* = 0.001, 0.008). Resuscitation including intubation at delivery was significantly higher in PDA ligation group (92.06% vs. 75.86%) with more incidence of metabolic acidosis (Ph < 7.2 or > 16) at initial sample. However, hydrocortisone use for hypotension < 1 week of life was more frequent in no PDA ligation group. The rest of clinical outcomes including mechanical ventilation, TPN duration and hospital days were significantly longer in PDA ligation group. Interestinly, with more weight gain and head circumference at discharge were shown in PDA ligation group, probably due to longer hospitalization (Table 1).

**Table 1:** Clinical characteristics of VLBWIs with hs PDA (*n* = 221)

*n* (%)	PDA ligation (*n* = 133)		No PDA ligation (*n* = 88)	*P*-value
Gestational age, week	27.02 (2.2)	27.98 (2.4)		0.001*
Birth weight, g	907.31 (258.4)	1006.07 (283.7)		0.008*
Male,	64 (48.1%)	31 (35.2%)		0.058
Antenatal steroid use	105 (79.0%)	67 (76.1%)		0.520
PROM ≥ 18 h	2 (1.5%)	0 (0.0%)		0.520
Maternal chorioamnionitis	44 (33.1%)	25 (28.4%)		0.520
Resuscitation including intubation at delivery	116 (92.1%)	66 (75.9%)		0.001*
Epinephrine use at delivery	10 (7.9%)	7 (8.1%)		0.980
Metabolic acidosis (Ph < 7.2 or > 16) at initial sample	34 (25.6%)	6 (6.8%)		0.001*
RDS	125 (94.0%)	84 (95.5%)		0.770
Severe RDS (≥ 2 surfactant)	44 (33.1%)	29 (33.0%)		0.980
Pneumothorax	14 (10.5%)	10 (11.4%)		0.850
Massive pulmonary hemorrhage	31 (23.3%)	15 (17.1%)		0.260
Hydrocortisone use for hypotension < 1 week of life	63 (47.4%)	58 (68.2%)		0.002*
Neonatal seizure	38 (28.6%)	18 (20.5%)		0.174
Mechanical ventilation, days	82.49 (50.3)	42.20 (32.1)		< 0.0001*
Hospital days	110.77 (55.8)	69.65 (44.4)		< 0.0001*
TPN duration, days	63.43 (36.2)	37.05 (27.5)		< 0.0001*
Hospital days	110.77 (55.8)	69.65 (44.4)		< 0.0001*
PDA related factors				
Mean postnatal days of PDA ligation	21.61 (19.65)	-		NA
Ibuprofen start, days of life	14.21 (5.19)	15.23 (5.39)		0.24
PDA ligation after medical treatment failure	29 (21.80%)	47(53.4%)		< 0.0001*

Values are means (standard deviation) or frequencies (percentage), as appropriate. *P* value < 0.05 is significant for χ [2] or Fisher’s exact test (categorical measures) and independent t test (continuous measures)

***Abbreviations***: VLBWI, very low birth weight infants; significant; hs, hemodynamically significant; PDA, patent ductus arteriosus; g, gram body weight; PROM, premature rupture of membrane; RDS, respiratory distress syndrome; IVH, intraventricular hemorrhage; TPN, total parental nutrition

Regarding hs PDA-related treatment, the incidence of PDA ligation following medical failure was 29 cases (21.80%). The 47(53.4%) infants in the no PDA ligation group demonstrated a response to ibuprofen, and surgical ligation was not pursued thereafter. Accordingly, the rest of 41 (18.5%) infants in the no PDA ligation group (*n* = 88) closed their hs PDAs with fluid restriction. Ibuprofen start day was not significantly different between the two groups The mean age (days of life) when PDA ligation was 21.6 days in PDA ligation group (Table 1).

The clinical outcomes manifested significantly worse outcomes in PDA ligation group. BPD and BPD ≥ moderate, sepsis, IVH (grade ≥ 3), PVL were notably higher in PDA ligation group. The mortality rate between the two groups was not significantly different (Table 2).

**Table 2:** Major clinical outcomes and Mortality; PDA ligation vs. no PDA ligation

	PDA ligation (*n* = 133)	No PDA ligation (*n* = 88)	*P*-value
BPD	121 (90.9%)	66 (75.0%)	0.001*
BPD ≥ moderate	109 (81.9%)	46 (52.3%)	< 0.0001*
Sepsis	43 (32.3%)	17 (19.3%)	0.033*
NEC (≥ grade 2)	12 (9.0%)	4 (4.5%)	0.435
NEC operation	5 (41.7%)	0 (0.0%)	0.184
IVH (grade ≥ 3)	43 (32.3%)	12 (13.6%)	0.002*
PVL	31 (23.3%)	21 (23.8%)	0.042*
ROP > stage 3	27 (21.1%)	9 (11.7%)	0.087
ROP laser treatment	23 (35.4%)	11 (36.7%)	0.505
Anti-VEGF	8 (12.3%)	2 (6.7%)	0.496
Mortality	2	1	0.116

Values are means (standard deviation) or frequencies (percentage), as appropriate. *P* value < 0.05 is significant for χ [2] or Fisher’s exact test (categorical measures) and independent t test (continuous measures)

***Abbreviations***: BPD, bronchopulmonary dysplasia; NEC, necrotizing enterocolitis; IVH, intraventricular hemorrhage; PVL, periventricular leukomalacia; ROP, retinopathy of prematurity; VEGF, vascular endothelial growth factor

### PDA ligation risk factor in a multivariable logistic regression analysis

PDA ligation risk factors were examined through a multivariable logistic regression analysis. Given the significance of gestational age and birth weight, resuscitation including intubation at delivery and IVH (grade ≥ 3) were identified as significant risk factors for PDA ligation in a multivariate analysis. Both Resuscitation including intubation at delivery and IVH (grade ≥ 3) were consistently associated an increased risk for PDA ligation (Table 3).

**Table 3:** PDA ligation risk factor in a multivariate logistic regression analysis

	Univariate		Multivariate	
	P	Odds ratio	P	Odds ratio
Gestational age, week	0.003*	0.829		
Birth weight, g	0.009*	0.999		
Resuscitation including intubation at delivery	0.001*	3.691	< 0.004*	1.728
Initial metabolic acidosis (Ph < 7.2 or > 16)	0.003*	2.387		
Neonatal seizure	0.176	1.556		
Sepsis	0.035*	0.501		
IVH (grade ≥ 3)	0.002*	3.026	0.029*	2.387
NEC (≥ grade 2)	0.216	2.085		
PVL	0.725	1.214		

Values are means (standard deviation) or frequencies (percentage), as appropriate. *P value < 0.05 is significant for χ [2] or Fisher’s exact test (categorical measures) and independent t test (continuous measures)

***Abbreviations***: PDA, patent ductus arteriosus; IVH, intraventricular hemorrhage; NEC, necrotizing enterocolitis; PVL, periventricular leukomalacia

At corrected age of 18–24 months, the 221 VLBWI who were diagnosed with hs PDA and followed up for neurodevelopmental assessment at corrected 18–24 months of age completed the cognitive, language and motor composites of the Bayley Scales of Infant and Toddler Development III. In the total population (*n* = 221), infants who underwent PDA ligation exhibited significantly lower cognitive scores compared to those who did not undergo surgical ligation. Subgrouping the neurodevelopmental assessment by gestational age as ≤ 26 weeks, > 26-≤28 weeks, and > 28-≤32 weeks further demonstrated that infants in the PDA ligation group still had significantly lower cognitive scores (Table 4).

**Table 4:** Neurodevelopmental outcomes of VLBWIs with PDA ligation at corrected 18–24 months of age on Bayley Scales of Infant and Toddler Development III (*n* = 221)

		PDA ligation (*n* = 133)	No PDA ligation (*n* = 88)	*P*-value
**Total** **(** ***n*** **= 221)**	**Score, mean ± SD**			
	Cognitive score	87.1 (15.8)	95.8 (14.0)	0.006*
	Language score	82.6 (18.1)	89.8 (14.9)	0.050
	Motor score	80.5 (17.7)	86.6 (18.7)	0.163
**≤ 26 weeks of GA**	**Score, mean ± SD**			
	Cognitive score	79.45 (20.3)	91.36 (18.6)	0.029*
	Language score	73.00 (17.7)	86.55 (20.1)	0.146
	Motor score	73.27 (20.2)	79.82 (18.3)	0.408
**> 26 to ≤ 28 weeks of GA**	**Score, mean ± SD**			
	Cognitive score	87.2 (13.7)	98.9 (10.4)	0.011*
	Language score	84.0 (15.6)	91.2 (11.2)	0.162
	Motor score	81.1 (16.6)	89.0 (19.6)	0.190
**> 28 to ≤ 32weeks of GA**	**Score, mean ± SD**			
	Cognitive score	89.2 (16.5)	97.9 (10.4)	0.026*
	Language score	85.0 (21.5)	92.6 (11.2)	0.384
	Motor score	83.3 (18.1)	93.6 (17.0)	0.198

Values are means (standard deviation) or frequencies (percentage), as appropriate. *P value < 0.05 is significant for χ [2] or Fisher’s exact test (categorical measures) and independent t test (continuous measures)

***Abbreviations***: VLBWI, very low birth weight infants; significant; hs, hemodynamically significant; PDA, patent ductus arteriosus; GA, gestational age

### Abnormal neurodevelopment risk factor in a multivariable logistic regression analysis

Abnormal neurodevelopmental risk factors were examined through a multivariable logistic regression analysis. Children were considered to have abnormal neurodevelopment if their scores were more than 2 standard deviations below the test mean (scores of < 80). Both IVH (grade ≥ 3) and PVL were consistently associated with an increased risk for abnormal neurodevelopment (Table 5).

**Table 5:** Univariate and Multivariate logistic regression analysis for Abnormal neurodevelopment^a^ at corrected 18–24 months of age on Bayley Scales of Infant and Toddler Development III at 18–24 months

	Univariate		Multivariate	
	P	Odds ratio	P	Odds ratio
Gestational age, week	0.052	0.939		
Birth weight, g	0.621	0.999		
Intubation at delivery	0.101	5.515		
Initial metabolic acidosis (Ph < 7.2 or > 16)	0.249	2.367		
Neonatal seizure	0.087	2.240		
Sepsis	0.794	1.121		
IVH (grade ≥ 3)	0.016*	3.079	0.020*	3.054
PDA liagtion	0.161	2.568		
NEC operation	0.794	0.667		
PVL	0.001*	3.375	0.001*	3.354

Values are means (standard deviation) or frequencies (percentage), as appropriate. *P value < 0.05 is significant for χ [2] or Fisher’s exact test (categorical measures) and independent t test (continuous measures)

^a^Children were considered to be at abnormal neurodevelopment if their scores were > 2 standard deviations below the test mean (scores of < 80)

***Abbreviations***: PDA, patent ductus arteriosus; IVH, intraventricular hemorrhage; NEC, necrotizing enterocolitis; PVL, periventricular leukomalacia

## Discussion

While previous studies have raised concerns about surgical ligation, we aimed to search several clinical risk factors of PDA ligation. Wu et al. [21] described that both medical and/or surgical treatment of hs PDA in extremely low birth weight (ELBW) were sicker at birth with a higher Clinical risk index for babies (CRIB) II score > 12, predicting a greater risk of neonatal mortality among this group of infants [22]. Base excess, a constituent of the CRIB II score, is an independent predictor of medical PDA closure response. (23–24) Further, those ELBW were associated with several worse short-term outcomes like BPD. Our study also similarly demonstrated that both resuscitation measures, including intubation at delivery, which suggests a sicker and more hypoxia-related event, and severe IVH with a grade of ≥ 3, consistently increased the risk for PDA ligation. As to clinical outcomes, PDA ligated group manifested significantly worse short-term hospital outcomes.

In regard to neurodevelopmental outcomes, the cognitive developmental delay was more found in PDA ligated VLBWI at corrected age of 18–24 months in our study. Kabra et al. [25] also found increased cognitive delay and a composite of NDI at 18-month follow-up in ligated compared with medically treated infants, after adjusting for perinatal covariates and the total dose of indomethacin received during hospitalization while another study also have determined surgical ligation as a higher risk factor for neurodevelopmental impairment as compared to medical treatment [26]. Janz-Robinson et al. [27] further reported that treatment with both medical and/or surgical treatment of PDA to be associated with neurodevelopmental impairment. Jhaveri et al. [28] additively reported improved neurodevelopmental outcomes in the delayed selective ligation group. Regarding risk factors for neurodevelopmental outcomes, infants who underwent PDA ligation exhibited significantly lower cognitive scores. However, only IVH (grade ≥ 3) and PVL were consistently associated with an increased risk for abnormal neurodevelopment at corrected 18–24 months of age, suggesting that PDA ligation itself did not increase the risk for abnormal neurodevelopment among our VLBWI with persistent hs PDA. After all, the risk of NDI associated with PDA ligation can be influenced by various prematurity related or predisposing factors, impact of anesthesia and postoperative hemodynamic compromise. The operative management of this condition encompasses procedures such as invasive mechanical ventilation, the requirement for analgesia, administration of induction and maintenance anesthetic agents, posterolateral thoracotomy, retraction of the left lung, and the application of a clip or ligature to the ductus arteriosus. Following the surgery, the compromised tolerance of increased left ventricular afterload due to the surgical interruption of the ductal shunt places infants at risk of acute left ventricular dysfunction. In our study, even though the cognitive developmental delay was consistently associated with VLBWI who underwent PDA ligation, we found that VLBWI who were sicker and more vulnerable, experiencing severe IVH (grade ≥ 3), consistently showed an increased risk for PDA ligation and abnormal neurodevelopment at corrected age of 18–24 months.

The present study has several limitations, including its single-center design and retrospective nature, which may not be optimal for establishing direct causal relationships. Further, advancements in clinical treatment for VLBWI beyond hs PDA treatment modalities may have occurred from 2015 to 2023. The study is also susceptible to the influence of various clinical conditions that may arise from prematurity itself. To assess neurodevelopmental assessment, we excluded the VLBWI who were not the followed-up at 18–24 months of age; resulting in a final inclusion of 221 VLBWI. It’s worth noting that hidden disabilities may have become apparent later than 18–24 months of age, and some infants might have significant developmental delays that were not categorized as impairments. Despite these limitations, the study’s strength lies in the observation of long-term neurodevelopmental outcomes using the Bayley Scales of Infant and Toddler Development III. Additionally, the study benefits from a large group of very low birth weight infants (VLBWI) in a single-center setting, allowing for the application of homogeneous treatment modalities for hemodynamically significant patent ductus arteriosus (hs PDA).

## Conclusion

In summary, our study showed that both resuscitation including intubation at delivery and IVH (grade ≥ 3) which reflects the vulnerability of VLBWI and consistently demonstrated an increased risk for PDA ligation. At corrected age of 18–24 months, the cognitive developmental delay was more found in PDA ligated VLBWI. Despite the consistent association between cognitive developmental delay and VLBWI who underwent PDA ligation, we observed that VLBWI who were sicker and more vulnerable, experiencing severe IVH (grade ≥ 3), consistently exhibited an increased risk for both PDA ligation and abnormal neurodevelopment at the corrected age of 18–24 months. Nonetheless, surgical ligation remains an important last stage of treatment modality. Infants with persistent symptomatic hs PDA suggests that the ductal shunt should not be neglected if medical therapy fails, and that surgical ligation remains an important treatment option for VLBWI.

## Data Availability

Availability of Data and Materials: The datasets used during the current study are available from the corresponding author upon request. Medical records are available in the Archive of the Department of Pediatrics of the Seoul St. Mary’s Hospital.
